# A Recent Advance in the Closure of Skin Wounds on Fragile Skin

**DOI:** 10.1155/2021/6687961

**Published:** 2021-06-26

**Authors:** John Ko, Jeffrey S. Freed

**Affiliations:** ^1^Department of Surgery, Icahn School of Medicine at Mount Sinai, 8409 Grand Avenue, Elmhurst, NY 11373, USA; ^2^Department of Surgery, Icahn School of Medicine at Mount Sinai, 969 Park Avenue, Ste. 1D, New York, NY 10028, USA

## Abstract

The delicate nature of the skin in elderly patients poses a difficult challenge to healthcare providers. Emergency departments are frequently presented with traumatic skin tears and soft tissue avulsions in this group of patients. Procedures aimed at closure of these types of wounds often result in worsening of the tears. The DermaClip® skin closure device, which can eliminate the need for anesthesia, addresses these disadvantages and allows for atraumatic, cosmetically satisfactory closure in a rapid and efficient manner, saving time, and costs.

## 1. Introduction

Emergency department healthcare providers are frequently faced with elderly patients presenting with a traumatic skin tear or soft tissue avulsion. Attempts at closure of these types of wounds often result in worsening of the tears or injury to the elevated flap because the skin and soft tissue are so fragile. Many methods have been devised but have been essentially unsuccessful.

A novel, new, noninvasive skin closure device, the DermaClip® skin closure device, addresses all the disadvantages and allows secure closure without further trauma to the compromised skin and soft tissue, with an excellent cosmetic result.

## 2. Case Presentation/Patient Information

Two patients, both elderly, presented with traumatic lacerations and were closed with DermaClip® devices. Both patients had age and comorbidity complications resulting in challenge closing fragile skin. The patients were closed by a single provider.

**Figure 1:**
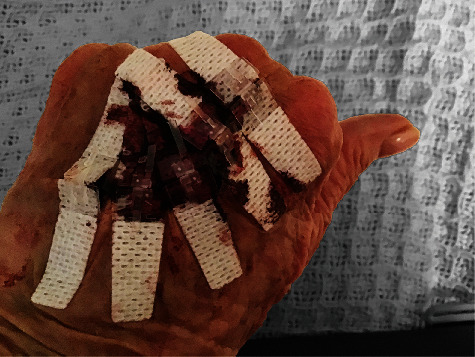
Patient 1, closed wound on day 1 after application of 5 Regular size DermaClip® devices.

Patient 1 was a 79-year-old female with fragile skin who presented with a traumatic flap laceration of the dorsum of her hand. She is anticoagulated for her heart valve replacement. The wound had been bleeding and untreated for over 24 hours prior to the patient presenting for treatment. The crescent shaped, full-thickness laceration was 7.5 cm in length with no debridement required. The skin tear was identified as ISTAP type 1, as the flap could be repositioned to cover the wound bed [1]. After cleaning the wound, the wound edges were reapproximated with 5 Regular size DermaClip devices (Figure 1). No anesthetic was used for this procedure.

**Figure 2:**
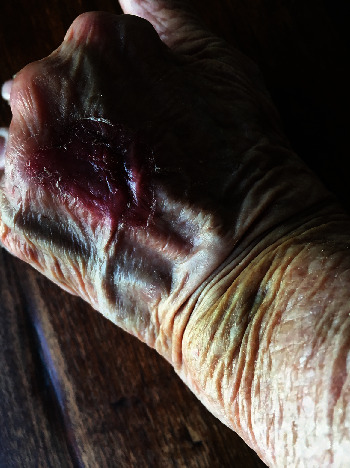
Patient 1, wound site on day 14 (~2 weeks) after injury.

**Figure 3:**
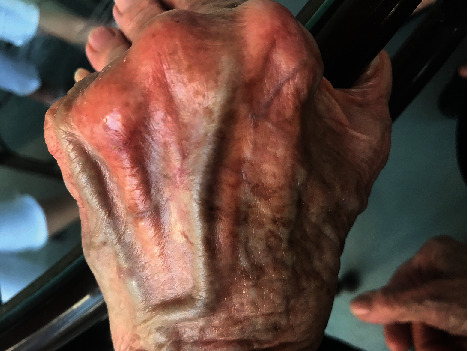
Patient 1, healed wound at day 135 (19 weeks) after injury.

**Figure 4:**
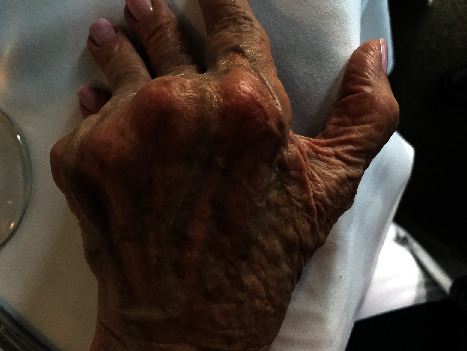
Patient 1, healed wound at day 289 (41 weeks) after injury.

Application of the DermaClip devices and closure of the wound were completed in less than 90 seconds. The wound progressively healed with the devices removed 12 days after closure with an excellent cosmetic result. Follow-up photographs were provided at 2 weeks (Figure 2), 19 weeks (Figure 3), and 41 weeks (Figure 4) after closure.

**Figure 5:**
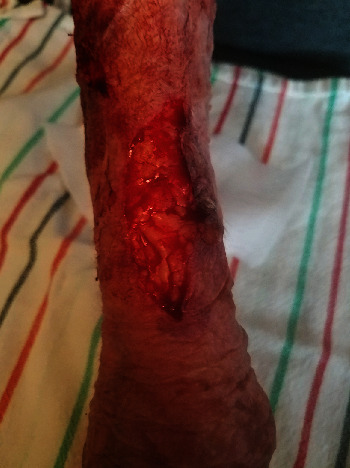
Patient 2, wound sites at time of injury before application of DermaClip® devices.

Patient 2 was an 84-year-old female with multiple myeloma on anticoagulants with thinning skin due to her age and long-term corticosteroid use. The patient had fallen outside onto the street, and her right arm had suffered traumatic lacerations with wide margins (Figure 5). The primary laceration was approximately 7.6 cm in length, and the secondary laceration was approximately 2.4 cm in length. The skin tear was identified between ISTAP types 1 and 2, as the expanse of the margins made it difficult to ascertain the extent of tissue loss.

After cleaning the wounds and stopping most of the bleeding with pressure, 5 Regular size DermaClip devices were placed on the primary laceration and 2 Regular size DermaClip devices were placed on the secondary laceration. Total time for closure of both wounds, including cleaning of the wound and application of pressure to slow bleeding, was 10 minutes, with device application and closure accounting for approximately 2 minutes of the total treatment time. No anesthetic was used, and the patient did not experience any discomfort during the closure.

**Figure 6:**
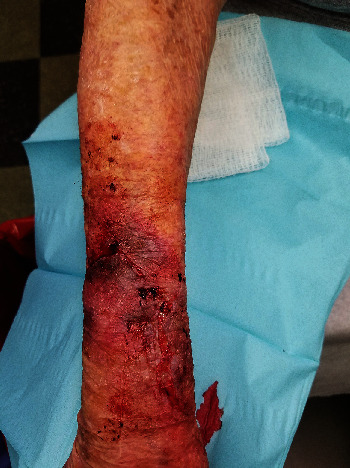
Patient 2, wound sites on day 8 (~1 week) at the time of the removal of devices.

**Figure 7:**
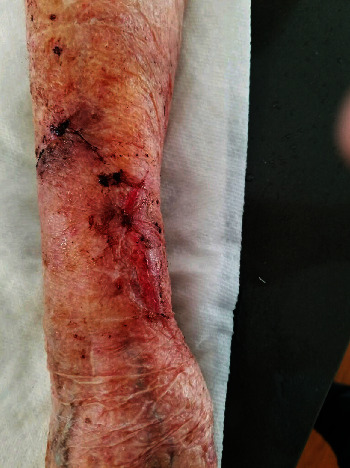
Patient 2, healing wound sites on day 15 (~2 weeks).

**Figure 8:**
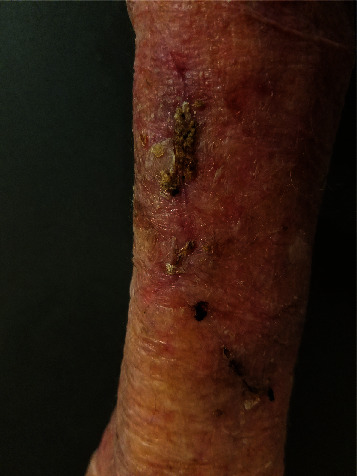
Patient 2, healing wound sites on day 50 (~7 weeks).

**Figure 9:**
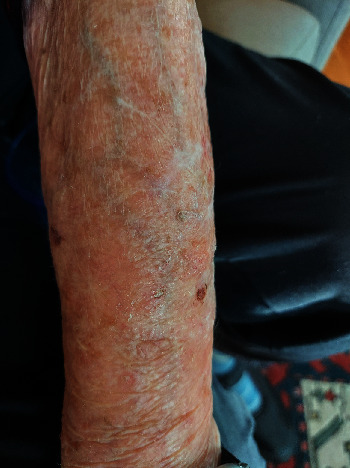
Patient 2, healing wound sites on day 153 (~22 weeks).

The devices were removed on day 8 (Figure 6), and the wound was examined again a week later, day 15 (Figure 7). At day 50 (Figure 8), the wound site showed no loss of tissue from either the trauma to or manipulation of skin when closing the wound. Additionally, the margins between the skin edges had closed, and significant healing progress was observed. A final follow-up photograph was provided at 22 weeks (Figure 9), where the scar was imperceptible.

## 3. Discussion

The rapid increase in the number of elderly individuals has, and increasingly will have, a significant impact on the need for healthcare providers who understand the unique requirements for the treatment of wounds in this population. A frequent wound for elderly patients, the skin tear is often underreported or misdiagnosed [1]. Estimates have shown that in the United States, there are approximately 1.5 million skin tears each year in institutionalized adults, potentially having prevalence rates equal to or greater than pressure ulcers [2, 3]. To deliver high-quality care that is cost-effective to this patient population, clinicians must provide care of wounds that considers the biologic and psychosocial complexities of aging skin and the vagaries of healing in general in this age group.

The fragility of the damaged skin in the aged results from physiologic changes and disease processes that are specific to these patients and other patients with certain underlying diseases and prior tissue-damaging therapy. As individuals age, the dermal layer of skin loses collagen and elastin, which then results in the thinning of the dermis and reduced protective mechanical function [4]. This is compounded by UV exposure, genetics, lifestyle, and the use of certain medications [5]. The fragility of aging skin renders it less able to repair itself [6].

Therefore, those patients most vulnerable to skin tears are generally also of advanced aged and are at the highest risk of developing infections and comorbidities. Treated improperly, these skin tears can evolve from uncomplicated wounds to become significant or complex wounds [1]. Given the high-risk nature of the skin and surrounding areas, it is important for the closure method itself not to cause additional trauma and to limit the potential for the wound area to become a complex, chronic wound and put additional strain on the patient and the healthcare system [7–9].

In addition, in patients with these skin conditions, the subcutaneous layer frequently has markedly decreased adipose tissue or has tissue of poor quality, creating a situation where this layer is functionally inadequate to support the injured skin layers. This is problematic for emergency room clinicians, who must struggle to close skin tears and flap lacerations where the surrounding skin holds sutures poorly, with a common outcome being the suture cutting through the skin. Additional trauma is created with each failed attempt [10]. These attempts at repair also often require firm closure for hemostasis, which further aggravates the potential for suture cutting of the skin and leads to prolonged healing times if not adequately apposed [11]. Also, the need to anesthetize the wound prior to closure carries the potential for hematoma development.

In typical closures, topical skin adhesives (TSAs) have been found to be more cost-effective than sutures in wounds up to 5 cm in length because of quick application and no follow-up, with the savings coming despite higher material cost of TSA (averaging $24 per ampule for wounds up to 5 cm in length or $4.80/cm) [12, 13]. Despite the frequency of use, skin tears are not always amenable to tissue adhesive closure due to the approximation complexities arising from the nature and shape of the wound, the tissue adhesive's inability to hold tension [14], and the length of the skin tear. Further, if the wound continues to ooze blood at the time of application of the TSA, the polymerization of the TSA can accelerate and the closure could be compromised [15]. Finally, TSAs are contraindicated in patients at higher risk of poor healing [16].

When caring for these injuries in patients in the aforementioned groups, the healthcare provider must consider these issues at all times. Multiple mitigation efforts have been developed in an attempt to address these issues. Simple sutures, sutures with bolsters, glues, retention devices, tapes, and horizontal mattress sutures have been placed to prevent skin tearing and allow effective wound closure [6]. However, all these measures have problems, including further tearing, burning secondary to chemical/thermal reactions, and further tissue trauma with necrosis. Additionally, many of these techniques require anesthesia, further increasing the chance of hematoma or tissue damage.

The DermaClip device allows the healthcare provider a solution for these issues. It is a noninvasive, needle-free alternative to sutures, staples, and TSAs that is composed of two pieces of adhesive joined by a polypropylene bridge. It requires usual skin disinfection but generally does not require anesthetic for application. The device is available in 2 sizes, regular and large, and is simple in design but advanced as a wound closure device. The material costs of DermaClip are lower than that of TSAs, as DermaClip devices can close wounds at an effective $3.67/cm, which represents a significant savings as compared to TSAs [13, 17]. DermaClip maintains the time and staffing benefits of TSA compared to suturing, including the following: single provider application, minimal time, limited need for anesthetic, minimal additional trauma to flap and potential for procedure-induced necrosis, limited need for removal procedure, minimal pain, and increased patient comfort [18]. It extends those benefits to wounds longer than 5 cm, wounds that have tension associated with the closure, and wounds that continue to ooze after closure. The DermaClip device deals with wound closure in an atraumatic, efficient, rapid, painless, and cost-effective manner (Table 1).

The device is applied to the approximated edges of a wound and is closed by pulling the polypropylene tabs in opposing directions until the device locks in the closed position. During closure, the angled faces of the polypropylene bridge encounter each other, thus lifting the wound edges. This lifting action puts the viable dermis on each side of the wound back into contact, creating a natural eversion of the skin edges on closure, relieving wound tension, promoting hemostasis, and avoiding the poor cosmesis and function often associated with an inverted scar.

It is left on and either removed or allowed to peel off after approximately 12-14 days, eliminating the follow-up office or emergency room visit, unless the patient has a concern. Also of note, the device can be applied by any healthcare extender, freeing the physician to deal with more complex patients.

The device has been used in multiple wound closure situations, including in the emergency room for lacerations in all age groups (geriatric through pediatric) and by surgical specialties for major surgical wound closures [19] and C-section closures [20]. The variety of wounds that can be rapidly, efficiently, and cosmetically closed is myriad. When debridement is not required, closure of simple wounds can generally be accomplished without anesthesia, unnecessary trauma from needle penetrations, or excessive wound tension. This allows device utilization in nonhospital or clinical settings, such as on-site at properly staffed long-term-care facilities or in conjunction with telemedicine platforms, avoiding transport of nonmobile patients when possible.

The wound closure experiences reflected in our two cases were very positive with respect to ease of use, patient comfort, and satisfaction. The DermaClip device addressed the difficulties in skin closure of the aging population with thinning skin. No anesthetic was required in either closure as the wound was able to be cleaned and closed without the patient experiencing pain during the procedure, although it is recognized that no debridement was required for either wound. With regard to the second patient, the performance of the device in effecting a good closure was unaffected by the continued oozing of the wound after closure. In both instances, an effective closure on a very difficult-to-close wound was accomplished in 10 minutes or less. In short, use of the DermaClip to effect the closures reviewed here allowed avoidance of potential complications that are commonly encountered when repairing fragile skin.

In summary, when dealing with the fragile skin of the elderly while attempting to close a tear or flap laceration, clinicians must take care to avoid introducing additional trauma while performing the repair. Limiting the introduction of foreign elements and extrinsic factors which may result in a hematoma or further disruption of the tissue can improve outcomes [21]. Therefore, any closure device that does not require skin manipulation, punctures, or embedding foreign materials while still effecting good closure is extremely valuable and should be considered for use on these patients. While these cases show the potential value of the DermaClip device for patients with skin complications, further studies should be undertaken to determine the full extent of the benefits of utilizing these types of atraumatic devices in the closure of fragile skin of elderly patients.

## 4. Patient Perspective

Both patients were followed until their wounds were completely healed. Patient 1 maintained hemostasis after the device was applied and during the entire course of wound healing. Both patients reported no additional discomfort during or after the closure without the use of a local anesthetic. The rapidity and ease of closure with the DermaClip devices, the absence of needles and anesthetic, and the avoidance of the potential skin shredding from sutures made the closures essentially uneventful. The cosmetic result was excellent with both patients having scars that were essentially imperceptible. Both patients expressed high levels of satisfaction with both the closure process and the results.

## 5. Conclusion

In repairing wounds in the elderly who have thinned, fragile skin, a device with the design and performance attributes of the DermaClip® wound closure device should be considered. The device is easily and rapidly deployed and rarely requires local anesthesia, which makes it cost-effective, time-, and resource-sparing, and especially useful in the emergency setting.

## Figures and Tables

**Table 1:** Attribute comparison for the DermaClip device.

	DermaClip	Sutures	Glues	ZipLine
Noninvasive & needle-free	✔		✔	✔
Creates wound edge eversion	✔	✔		
Disperses wound tension	✔			✔
No pain or burning associated with application	✔			✔
Breathable	✔	✔		
Clean application, no mess	✔			✔
Time-effective	✔		✔	✔
Cost-effective (excl. cost of time)	✔	✔	✔	

## Data Availability

The authors confirm that the data supporting the findings of this study are available within the article, its figures, and its references.
